# Association of androgen receptor GGN repeat length polymorphism and male infertility in Khuzestan, Iran

**Published:** 2015-05

**Authors:** Mohamad Moghadam, Saied Reza Khatami, Hamid Galehdari

**Affiliations:** *Department of Genetics, Faculty of Sciences, Shahid Chamran University, Ahvaz, Iran.*

**Keywords:** *Androgen receptor*, *GGN repeat*, *Male infertility*, *Khuzestan*

## Abstract

**Background:**

Androgens play critical role in secondary sexual and male gonads differentiations such as spermatogenesis, via androgen receptor. The human androgen receptor (*AR*) encoding gene contains two regions with three nucleotide polymorphic repeats (CAG and GGN) in the first exon. Unlike the CAG repeats, the GGN has been less studied because of technical difficulties, so the functional role of these polymorphic repeats is still unclear.

**Objective:**

The goal of this study was to investigate any relationship between GGN repeat length in the first exon of *AR* gene and idiopathic male infertility in southwest of Iran.

**Materials and Methods:**

This is the first study on GGN repeat of *AR* gene in infertile male in Khuzestan, Iran. We used polymerase chain reaction (PCR) and polyacrylamide gel electrophoresis to categorize GGN repeat lengths in 72 infertile and 72 fertile men. Afterwards we sequenced the PCR products to determine the exact length of GGN repeat in each category. Our samples included 36 azoospermic and 36 oligozoospermic men as cases and 72 fertile men as control group.

**Results:**

We found that the numbers of repeats in the cases range from 18 to 25, while in the controls this range is from 20 to 28. The results showed a significant relation between the length of GGN repeat and fertility (p=0.015). The most frequent alleles were alleles with 24 and 25 repeats respectively in case and control groups. On the other hand no significant differences were found between Arab and non-Arab cases by considering GGN repeat lengths (p=0.234).

**Conclusion:**

Due to our results, there is a significant association between the presence of allele with 24 repeats and susceptibility to male infertility. Therefore this polymorphism should be considered in future studies to clarify etiology of disorders related to androgen receptor activity.

## Introduction

Approximately 15-18% of couples are affected with infertility, which in 50% of these couples, male factor is responsible. Genetic components are major reasons for male infertility in spite of environmental factors. Failure in spermatogenesis is one of the common causes of infertility in men. Androgens, acting through the androgen receptor (AR), play a role in secondary sexual differentiation from the prenatal stages to the adulthood, including spermatogenesis (1). AR is fundamental for differentiation of the external genitalia and prostate during development. It is also responsible for the changes that occur at puberty in males and maturation of spermatides and their differentiation (2). AR has three functional domains: N-terminal transactivating domain, DNA binding domain and C-terminal hormone binding domain. Transactivating domain is the most polymorphic domain in AR, because of two polymorphic amino acid tracts: polyglutamine tract and polyglycin stretch (3).

*AR* gene, including eight exons, is located on Xq 11-12 (4). The gene has two polymorphic sites in the first exon (5, 6). One of them is a long polymorphic trinucleotide (GGN) repeats with a common sense codon sequence of GGN tract (GGT)_3_ GGG (GGT)_2_ (GGC)_4-25_ which encodes polyglycin. The other polymorphic repeat is (CAG) _9-34_ CAA that is called CAG repeat and locates in 5' portion of the first exon and it is responsible for encoding polyglutamine tract. Although CAG repeat length has been well studied in different populations but the data about GGN repeat is still incomplete. The number of CAG and GGN repeat ranges from about 10-35 (with a mean of 20-23) and 10-30 (with a mean of 20-21) respectively in normal men (7). CAG repeat regulates the transcriptional activity of AR protein (8). 

Longer CAG repeat lengths cause reduced AR transcriptional activity both in vivo and in vitro, and it is clear that there is an inverse correlation between CAG number and androgenicity (9). Because of technical difficulties, GGN repeats have been sequenced rarely and unlike CAG repeat that has been the subject of many studies, these repeats are less examined. However there is in vitro evidence that proves glycine repeats have role in trans activity of AR protein, and deletion of GGN repeat reduces AR activity by ~30% (7, 10). Due to difficulties of GC-rich regions PCR amplification, investigation in GGN trinucleotide repeats is relatively less than CAG repeats, and its exact function is still unclear. The association between GGN and infertility was examined in few studies. 

Tut *et al* reported no significance difference in distributions of GGN repeat lengths between infertile men and healthy individuals (9). Lundin *et al* found that distributions of the 23 and 24 GGN repeat alleles did not differ significantly between infertile men with sperm count <5×10^6^ ml and controls (3). Ferlin *et al*, Ruhayel *et al*, Rajendar *et al*, Akinloye *et al*, and Nallar *et al* did not observe any differences in GGN repeat length between fertile and infertile men (1, 5, 6, 11, 12). In some of these studies the mutual effect of both repeats on male infertility was studied; "protective" and "at risk" CAG/GGN haplotypes were determined. The results showed the “protective” and the “at risk” CAG/GGC haplotypes were different (5, 6). 

Despite the important role of GGN repeats, there are a limit number of studies on them; due to our knowledge no study has been done to determine the role of GGN repeats in male infertility in Iran. Therefore, we decided to investigate the GGN repeat lengths in Khuzestan, the southwest province of Iran, in order to find any relationship between the length of these repeats and idiopathic male infertility.

## Materials and methods

According to the Cochrane sample size formula, 72 idiopathic infertile men (Arab or non-Arab), ranged between 33 to 39 years and less than 15×10^6 ^spermatozoia in 1ml of their semen admitted to the Imam Khomeini hospital and Shafa hospital of Ahvaz, Khuzestan, Iran were enrolled in our case control study. Cases with male accessory gland infections, hypogonadotropic hypogonadism, clinical varicocele, rectractile testis, genital trauma, drug consumption or concomitant hormonal treatment and Y chromosome microdeletions (investigated in three regions (AZFa, AZFb, and AZFc) by six Sequence Tagged Sites (STS) markers) were excluded.

The 72 samples were divided into two groups based on sperm count parameters: 36 individuals with azoospermia (absence of spermatozoia in their semen) and 36 individuals with oligozoospermia (<15×10^6^ spermatozoia in 1 ml). 72 men, ranged between 32 to 38 years, who had at least one child, were included in our study as the control group. All participants gave their informed consent prior to their inclusion into the study with confirmation of research council of genetics department.

DNA extracted from peripheral blood cells using standard technique. Polymerase chain reaction (PCR) using following primers amplified GGN segment: F: 5'-AGAAGGCCA GTTGTATGGACC-3' and R: 5'-TGGGATAGG GCACTCTGCTCA-3'. Whereas this PCR product is a GC rich DNA fragment, the standard PCR and use of common additive such as DMSO, Formamide, Betain, etc. were unable to amplify this region. We established a new protocol by using 100µM 7-deazaguanine, 2 M Betaine (SIGMA ALDRICH, USA), 200 μM dATP, 200 μm dCTP, 200 μM dTTP (BIORON GmbH, Germany), 2mM MgCl_2_,100 μM dGTP 1x GC PCR buffer and 2 unit LA-Taq (TaKaRa Bio Inc, Japan).

We used 0.75 pM of each designed primers and 300 ng sample DNA in a total of 25µ reaction mixture. This PCR consisted of an initial denaturation step of 5 min at 94^o^C, followed by 30 cycles of 1 min denaturation at 94^o^C, 45 sec annealing at 62^o^C and extension step at 72^o^C for 30 sec and 7 min final elongation was performed at 72^o^C. The PCR products were run on 12% polyacrylamide gel and were sub grouped according to product length. One PCR product was selected from each group and was sequenced in order to determine GGN repeat size.

**Statistical analysis**

The statistical significance of the results between different groups, due to the abnormal distribution of GGN repeat lengths, were calculated using Mann-Whitney U test by Statistical Package for the Social Sciences, version 16.0, SPSS Inc., Chicago, Illinois, USA.

## Results

**Figure 1:**
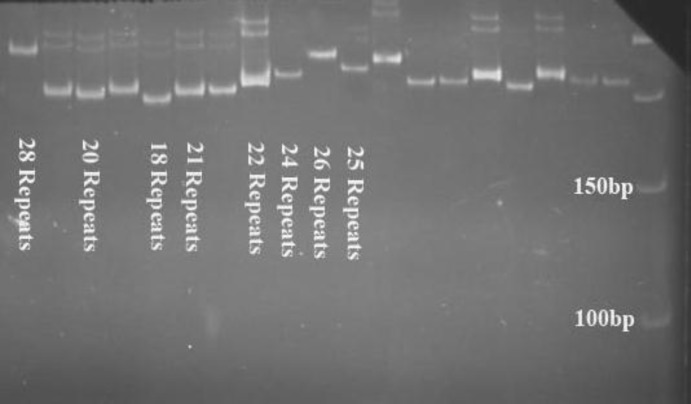
Categorizations of GGN repeat lengths using polyacrylamide gel electrophoresis. The number of GGN repeats ranged from 18-27 and from 20-28 respectively in infertile and fertile men

**Figure 2:**
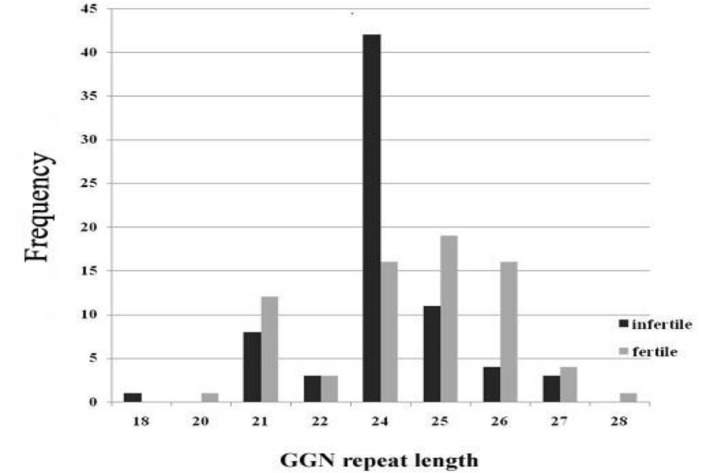
Distribution of GGN allele repeats in cases and controls. The number of studied participants with a particular number of GGN repeat in their *AR* gene is shown versus the length of GGN repeats. The allele with 24 and 25 GGN repeats were the most frequent alleles in infertile and fertile men, respectively. The number of GGN repeats ranged from 18-27 in infertile men, and from 20-28 in healthy men. As the chart shows, allele with 18 GGN repeats has been absent in control groups and allele with 20 GGN repeats has not been found in patient group

Our findings showed that the number of GGN repeats ranged from 18-27 and from 20 to 28 respectively in infertile and fertile men. Figure 1 is a representative of all gel electrophoresis. Each of the alleles with 18, 20, and 28 repeats was observed in one individual, so most of alleles ranged from 21 to 27 repeats. The statistical analysis revealed that GGN repeat lengths differed significantly among cases and controls (p=0.015). Cases were distributed in seven different GGN alleles and most common GGN allele had 24 repeats, whereas eight GGN alleles were found in control group with the most frequent allele of 25 repeats (Figure 2). 

Alleles with 20 and 28 repeats, which are present in two normal individuals, are absent in case group. In the other hand allele with 18 repeats was absent in control group. Although more than 58% of infertile individuals had the same GGN repeat lengths of 24 repeats, the distribution of GGN repeat lengths were far more symmetrical in control group. Due to our data, there was a strong correlation between allele with 24 repeats and infertility (p=0.015. The allele frequencies are shown in table I. 

Since ethnic origin play a major role in function of many polymorphisms, our participants were divided into two groups, Arab and non-Arab, according to their ethnicity. As *AR* gene is located on X chromosome our cases were divided based on their mother's ethnicity. We found no significant differences between Arab and non-Arab groups (p=0.234). AR alleles in all participants distributed in 9 alleles in the Khuzestan population. Most common alleles among all samples were alleles with 24 and 25 GGN repeats (40.2% and 20.8% respectively). 

**Table I:** The allele frequencies in different categories

** Repeats**	**Number of alleles **		**Total**
	**Case**	**Control**	
18	1	0	1
20	0	1	1
21	8	12	20
22	3	3	6
24	42	16	58
25	11	19	30
26	4	16	20
27	3	4	7
28	0	1	1
Total	72	72	144

* Repeats fertility Cross tabulation

## Discussion

There are many studies on *AR* gene as a responsible factor for spermatogenesis failure, but the role of polymorphic repeats in male infertility is unclear.

In this study we analyzed GGN repeat lengths in 72 idiopathic infertile men and 72 fertile men. We found that alleles with 24 and 25 GGN repeats are the most common alleles in infertile and fertile men, respectively. Other studies have revealed that dominant alleles have been 23 and 24 GGN repeats in Indian population and 21 and 22 GGN repeats among Italian men (1,5). We found a significant association between *AR* gene GGN repeat lengths and infertility in Iranian men; while previous studies on GGN repeat lengths in other population showed no association with infertility while a study on Chinese infertile men revealed association of shorter GGN repeats with idiopatic infertility(13,14). This controversy could be explained via the principle that an identical polymorphism may have different effect on different ethnicities. The predominant allele in our infertile group was the allele with 24 repeats (58.3%) while the frequency of this allele in fertile group was 22.2%. This shows a significant association between the presence of allele with 24 repeats and susceptibility to male infertility. 

In addition to this result of our study there is another study which shows a probable contribution of GGN 24 allele to AR dysfunction, which is in correlation with cryptorchidism and spermatogenic failure (12). Studies based on GGN repeat lengths in other diseases, have shown different results: Some studies on prostate cancer have detected no association and others have reported a correlation between shorter GGN repeats and higher risk of prostate cancer (15-20). Such contradictory results also have been observed in studies on cryptorchydism and hypospodias (21-23). Moreover, distinct findings have been announced for breast cancer (24-26). Complete androgen insensitivity syndrome and hypospodias were associated with longer alleles of GGN and CAG repeats (27). On the other hand, shorter GGN repeats could also increase the rate of recurrent spontaneous abortion (28). Therefore the role of the GGN repeat in AR function is still unclear and more investigations are required.

Since ethnical differences may affect the phenotypes resulted from an identical polymorphism, we investigated GGN repeat numbers in our case and control groups according to ethnicity. We determined GGN repeat lengths in Arab and non-Arab fertile and infertile men (mean of GGN in Arab infertile men: 23.67±0.316; non-Arab infertile men: 24.05±0.221; Arab fertile men: 24.37±0.373; non-Arab fertile men: 24.24±0.289). The most frequent GGN repeat lengths in Arab fertile men were 24 and 25 but the most common alleles in non-Arab fertile men were alleles with 25 and 26 GGN repeats. 

On the other hand, the most prevalent GGN repeat length in Arab and non-Arab infertile men, was the allele with 24 repeats. Our finding showed that GGN repeat numbers have no significant difference between Arab and non-Arab men in fertile and infertile groups.

 Androgens are known to play role in spermatogenesis and sperm differentiation, therefore alterations in androgen receptor could affect spermatogenesis (29). 

There are two statements that demonstrate how GGN repeat lengths could affect AR function. The first one explains how shorter GGN repeats can impair ligand binding and have negative effect on androgen dependent tissues (30). The second statement demonstrates that any alterations in GGN repeat lengths can change mRNA folding. These changes could affect AR mRNA translation and desensitize cells to androgens (23,31,32). It seems that our data of relationship between shorter GGN repeats and infertility confirms these statements.

## Conclusion

In conclusion, the GGN repeat length in *AR* gene can affect the risk of men infertility. However more in vivo and in vitro investigations are required to reveal the role of GGN repeat polymorphism in androgen receptor activity, specifically allele with 24 repeats should considered.
